# Deprivation Has Inconsistent Effects on Delay Discounting: A Review

**DOI:** 10.3389/fnbeh.2022.787322

**Published:** 2022-02-10

**Authors:** Haylee Downey, Jeremy M. Haynes, Hannah M. Johnson, Amy L. Odum

**Affiliations:** ^1^Odum Laboratory, Department of Psychology, Utah State University, Logan, UT, United States; ^2^Translational Biology Medicine and Health Graduate Program, Virginia Tech, Blacksburg, VA, United States

**Keywords:** delay discounting, review, state, deprivation, withdrawal

## Abstract

Delay discounting, the tendency for outcomes to be devalued as they are more temporally remote, has implications as a target for behavioral interventions. Because of these implications, it is important to understand how different states individuals may face, such as deprivation, influence the degree of delay discounting. Both dual systems models and state-trait views of delay discounting assume that deprivation may result in steeper delay discounting. Despite early inconsistencies and mixed results, researchers have sometimes asserted that deprivation increases delay discounting, with few qualifications. The aim of this review was to determine what empirical effect, if any, deprivation has on delay discounting. We considered many kinds of deprivation, such as deprivation from sleep, drugs, and food in humans and non-human animals. For 23 studies, we analyzed the effect of deprivation on delay discounting by computing effect sizes for the difference between delay discounting in a control, or baseline, condition and delay discounting in a deprived state. We discuss these 23 studies and other relevant studies found in our search in a narrative review. Overall, we found mixed effects of deprivation on delay discounting. The effect may depend on what type of deprivation participants faced. Effect sizes for deprivation types ranged from small for sleep deprivation (Hedge's *g*s between −0.21 and 0.07) to large for opiate deprivation (Hedge's *g*s between 0.42 and 1.72). We discuss possible reasons why the effect of deprivation on delay discounting may depend on deprivation type, including the use of imagined manipulations and deprivation intensity. The inconsistency in results across studies, even when comparing within the same type of deprivation, indicates that more experiments are needed to reach a consensus on the effects of deprivation on delay discounting. A basic understanding of how states affect delay discounting may inform translational efforts.

## Introduction

Delay discounting refers to the tendency for outcomes to be devalued as they occur more remotely in the future (Mazur, 1987; Odum, 2011a). Delay discounting is used as a measure of sensitivity to delayed consequences, where greater delay discounting indicates less sensitivity to delayed consequences (Strickland and Johnson, 2021). Greater degree of delay discounting has been associated with a variety of poor health behaviors, including smoking (e.g., Bickel et al., 1999; Mitchell, 1999), substance use (e.g., Reynolds, 2006; MacKillop et al., 2011), more energy-dense food purchasing choices (e.g., Appelhans et al., 2019), risky sexual behaviors (e.g., Johnson and Bruner, 2012; Sweeny et al., 2020), problematic gambling (e.g., Alessi and Petry, 2003), and lower exercise frequency (e.g., Daugherty and Brase, 2010; Sweeney and Culcea, 2017). In addition, a meta-analysis indicated that individuals diagnosed with schizophrenia, bipolar disorder, and major depressive disorder may tend to have steeper delay discounting than controls (Amlung et al., 2019). Delay discounting also predicts success in substance use treatment programs for adolescents using marijuana (Stanger et al., 2012) and for mothers who smoke tobacco cigarettes (Yoon et al., 2007). Because of associations with numerous health behaviors and psychiatric illnesses (Amlung et al., 2019; Levitt et al., 2020), delay discounting has been called a trans-disease process (Bickel et al., 2019; Felton et al., 2020; although see Bailey et al., 2021).

As a trans-disease process, delay discounting may be so steep or so shallow that it is considered maladaptive. For instance, individuals with substance use disorders may show excessive delay discounting (i.e., less sensitivity to delayed rewards) whereas individuals with anorexia nervosa may show especially low delay discounting (i.e., less sensitivity to immediate rewards; Levitt al., 2020). Several behavioral interventions have been developed that seek to reduce steep discounting, and thus patterns of maladaptive behavior (Rung and Madden, 2018). For instance, episodic future thinking (EFT; prospective imagining) has been shown to reduce delay discounting of money and number of self-administered cigarette puffs in the laboratory (Stein et al., 2016). To help individuals make optimal choices (i.e., choices that decrease risk of morbidity and mortality; Fields et al., 2014), it is important to consider the state that a person is in while making a choice. Delay discounting may change due to changes in state (Odum and Baumann, 2010). Deprivation is a state that may influence sensitivity to rewards. One might reasonably predict that individuals are more sensitive to immediate rewards when they are hungry, tired, thirsty, or more broadly, when they are deprived of something they need.

Deprivation is generally regarded as a fundamental determinant of reinforcer effectiveness, especially for behavior analysts (e.g., Michael, 1982; Miller, 2006). For instance, food may be more valuable when an individual is hungry and less so when sated. Furthermore, non-human animals are generally food restricted in behavioral research when food serves as a reinforcer (e.g., Hurwitz and Davis, 1983). Evolutionarily, it may be *adaptive* for immediate outcomes to be more valuable when deprived (Logue, 1988). Withdrawal, or deprivation from a drug, may increase valuation for immediate rewards specifically when the reward may be used to reduce negative affect brought on by withdrawal (Baker et al., 2004). Deprivation clearly has implications for the valuation of an outcome; after being deprived, something one needs immediately to survive may have a much higher value than other things (see Loewenstein, 1996).

The relationship between deprivation and valuation was studied as early as the 1980s in the self-control paradigm (e.g., Christensen-Szalanski et al., 1980). In the self-control and the delay discounting paradigms, participants make a series of choices between smaller sooner and larger later outcomes. In the delay discounting paradigm, tasks aim to find amounts participants are indifferent to receiving now or at a range of delays (Odum, 2011a). Indifference points are then plotted to create a delay discounting curve and mathematical models can be fit to the indifference points (see, e.g., Mazur, 1987; Green and Myerson, 2004). The dependent measures often used in delay discounting, the parameter *k* and the Area Under the Curve (AUC), are determined by the shape of the whole curve. In delay discounting, a greater number of smaller sooner choices results in a steeper delay discounting curve. In contrast, there are no indifference point curves in the self-control paradigm. Rather, the frequency of larger later choices may be determined for a number of delays or sometimes only one delay (Evenden and Ryan, 1996). A greater number of choices for larger later outcomes indicates more self-control and less impulsivity (De Wit, 2009). In humans and non-human animals, number of choices for larger later outcomes has been found to found to increase, decrease, and not change as a result of food deprivation (Logue and Peña-Correal, 1985; Logue et al., 1988; Kirk and Logue, 1997), contrary to assumptions. Other frameworks that predict an increase in sensitivity to immediate consequences due to deprivation include dual systems approaches (e.g., Van den Bos and McClure, 2012).

Delay discounting has long been theorized to involve the interplay between two (dual) systems (e.g., Schneider and Shiffrin, 1977; Thaler and Shefrin, 1981; Schelling, 1984). Some researchers conceptualize impulsivity as transitioning from cold to hot states (Logue, 1988; Metcalfe and Mischel, 1999; Frederick et al., 2002) while others refer to a myopic “doer” and a farsighted “planner” (Thaler and Shefrin, 1981). More recently, researchers have investigated how several different neurological systems may interact to determine delay discounting choices (Frost and McNaughton, 2017; Noda et al., 2020; Loganathan et al., 2021). These models all include a valuation system and a cognitive control system. The valuation system consists of at least the ventral striatum, ventromedial prefrontal cortex, and medial orbitofrontal cortex and *determines* the present value of the two choice alternatives (smaller sooner and larger later, e.g., Noda et al., 2020; Loganathan et al., 2021; Stanger et al., 2013). The cognitive control system, including the lateral prefrontal cortex and dorsal anterior cingulate cortex, *compares* the present value of the two choices (Bickel et al., 2018; Noda et al., 2020; Loganathan et al., 2021).

In the competing neurobehavioral decision systems (CNDS) dual-systems model, dysregulation of the cognitive control system and the valuation system leads to maladaptive behavior (Bickel et al., 2012, 2016, 2019). Greater activation of the valuation system relative to the control system is associated with more choices for smaller sooner outcomes in delay discounting tasks (Frost and McNaughton, 2017). For example, a smaller Area Under the Curve (AUC; Myerson et al., 2001) is associated with greater activation in the valuation system, specifically the ventral striatum, and less activation in the executive system, specifically the ventromedial prefrontal cortex (Frost and McNaughton, 2017). Dysregulation of the executive and valuation systems is thought to be caused by factors such as stress and substance use (e.g., cocaine, Bickel et al., 2016). For instance, stress may reduce cognitive resources, leading to a hypoactive control system (Bickel et al., 2014, 2016). Accordingly, the CNDS model predicts that deprivation may result in hyperactivity in the valuation system or hypoactivity in the control system, leading to a greater number of choices for smaller sooner outcomes in a delay discounting task, seemingly resulting in greater sensitivity to immediate outcomes (Loewenstein, 1996; Bickel et al., 2012; Van den Bos and McClure, 2012).

Because delay discounting may be both state-like and trait-like (Odum, 2011b; Odum et al., 2020; Haynes et al., 2021), one may also predict that deprivation can modulate delay discounting. Trait influences on delay discounting are evidenced by the fact that delay discounting measurements for individuals tend to be relatively stable over time and relatively similar in different situations (Odum and Baumann, 2010; Felton et al., 2020). State effects occur when delay discounting differs across repeated measurements due to changes in the environment or organism. For example, in one experiment, 20 individuals with problematic gambling completed delay discounting tasks in a gambling setting (i.e., a betting facility with a bar) or at a non-gambling setting (e.g., a coffee shop; Dixon et al., 2006). Individuals tended to have a lower AUC (steeper delay discounting) when they completed the task in the gambling setting compared to the non-gambling setting, demonstrating that context may play a role in determining degree of delay discounting. Drug administration (De Wit and Mitchell, 2010), emotion (Wilson and Daly, 2004), stress (Fields et al., 2014), blood glucose level (Wang and Dvorak, 2010; Wang and Huangfu, 2017), and context (Dixon et al., 2006) have all been investigated as states that may influence delay discounting. Because several state manipulations have been shown to modulate delay discounting, it is reasonable to predict that delay discounting may change due to deprivation manipulations as well.

In sum, deprivation has generally been thought to result in increased impulsivity, an assumption with arguably high face validity. However, it is not clear exactly *how* deprivation (and other experimental manipulations) may result in changes in delay discounting (Bailey et al., 2021). Although it seems clear that valuation of outcomes may change due to deprivation, there may not necessarily be a direct impact on the process of delay discounting itself. It may be that deprivation changes subjective valuation, which may systematically influence choices on a delay discounting task (and thus *k-*values), but the underlying *process* of discounting delayed rewards and sensitivity to delayed outcomes may remain the same.

In addition to underdeveloped theoretical explanations, results of early experiments on the effect of deprivation on delay discounting are mixed (e.g., Richards et al., 1997; Giordano et al., 2002; Mitchell, 2004). Researchers have often concluded that deprivation magnifies impulsivity, generally citing two experiments that reported large increases in delay discounting (i.e., Giordano et al., 2002; Field et al., 2006; see, e.g., Berns et al., 2007; De Wit, 2009; Van den Bos and McClure, 2012; Ashare and Kable, 2015; see however Bickel et al., 2015). Because studies that have shown little to no change in delay discounting due to deprivation may not have been cited as frequently as those that report large changes, the effects of deprivation may not be as clear as is commonly represented. Therefore, we conducted a review of experiments that measured delay discounting and manipulated deprivation level in human and non-human animals. For studies with available data, we computed and compared effect sizes. We discuss other relevant studies in a narrative review.

## Method

### Literature Search and Screening

**Figure 1:**
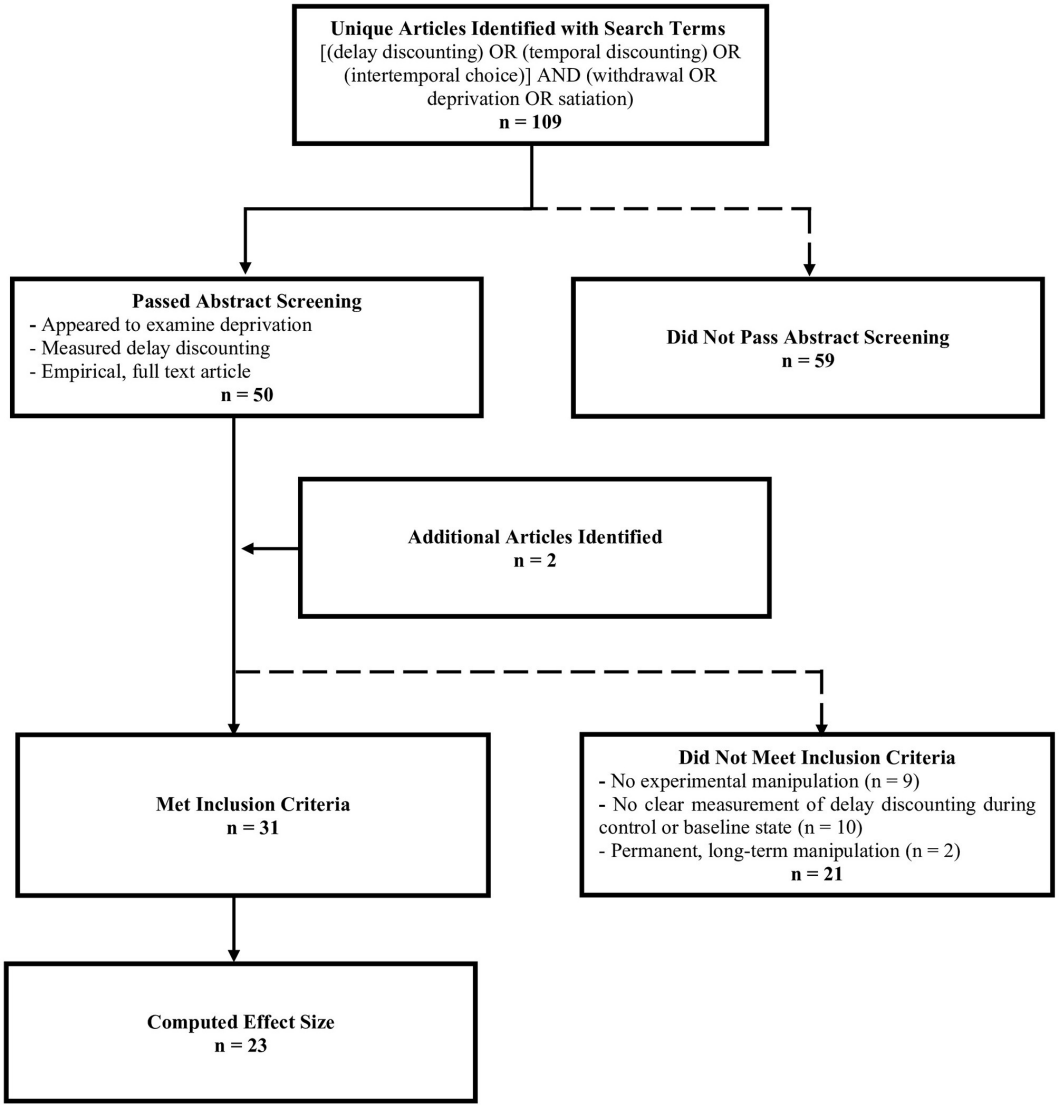
Number of articles included at each stage of the screening process. Dashed lines indicate points at which articles were excluded.

We searched PubMed and EBSCOhost to identify studies that assessed the effect of withdrawal or deprivation on delay discounting. The original search was conducted in September 2019 using the terms (“delay discounting” or “temporal discounting” or “intertemporal choice”) and (“deprivation” or “withdrawal” or “satiation.” Additional searches were conducted in June 2021 to include any more recently published articles. The searches resulted in a total of 109 unique articles. Abstracts were screened to ensure studies were relevant, empirical, and measured delay discounting. A total of 50 articles passed abstract screening. We included two additional articles that were not found in the literature search; these articles were found during manuscript preparation or in the references of articles that passed screening and were relevant to the review. Additional criteria were imposed to compute and compare effect sizes. Some articles did not clearly measure delay discounting during a deprivation state and a control state or baseline state and were thus excluded (*n* = 10). Studies that did not experimentally manipulate deprivation were also excluded (*n* = 9; e.g., studies that used self-reported deprivation as a covariate). Non-human animal rearing experiments (e.g., rats reared in social isolation; *n* = 2) were excluded because these studies were studying phenomena that are arguably different from the purpose of the review, which was to examine short-term state changes in deprivation state A total of 31 studies met inclusion criteria (see Figure 1).

### Data Collection

Three authors extracted data from articles that met inclusion criteria. To compute effect sizes, we collected the sample size of each experimental group and measures of central tendency and variability for delay discounting for each study. If measures of central tendency and variability were not available, we used the result of a *t*-test or Cohen's *d*. We did not compute effect sizes for studies that solely reported an *F*-statistic because effect sizes may be inflated when calculated from *F*-statistics (Hullett and Levine, 2003). The data we collected were listed in the text or Supplementary Material, represented in a figure, or provided by an author. If data were in a figure, a graphical data extraction tool was used to estimate the measure (Rohatgi, 2018). If the data were not present in the article and the study was published in the last 10 years, the corresponding author was contacted via email, once initially and once a month later to follow up if necessary. We contacted (or attempted to contact) authors of 6 articles. We were able to compute 54 effect sizes from 23 studies. Studies that used *k* as a dependent measure were reverse coded to aid in interpretation; AUC and *k* are inversely related, so reverse coding *k* results in similar interpretation for the two measures. For studies without available effect size data, we discussed the study in a narrative review if the study conducted an experiment on the effect of deprivation on delay discounting and at least discussed the result of the manipulation.

### Computation of Effect Sizes

To estimate the effect of deprivation on delay discounting, we calculated Hedge's *g* for each study with data available and that met our inclusion criteria. Hedge's *g* is a measure of effect size, calculated from Cohen's *d*, that corrects for an upward bias in effect size among small samples (*N* < 20; Goulet-Pelletier and Cousineau, 2018). One study reported only Cohen's *d* and no descriptive statistics (Skrynka and Vincent, 2019); however, for all other studies, we calculated Cohen's *d* from descriptive statistics reported in the text or obtained from the authors, and from *t*-statistics. For studies that reported descriptive statistics, we calculated Cohen's *d* using Equation (1),


(1) $$\begin{array}{l} \text{Cohen’s d}=\frac{M_{Non\;-\;Deprived}\;-\;M_{Deprived}}{Pooled\;SD} \end{array}$$

where *M*_*Non*−*Deprived*_ and *M*_*Deprived*_ are the mean estimates of delay discounting (e.g., AUC) obtained from the non-deprived and deprived groups, respectively, and pooled *SD* is the pooled standard deviation of the estimates of delay discounting. For between-subject designs, the pooled *SD* was calculated using Equation (2),


(2) $$\begin{array}{l} {Pooled\;SD}_{Between}\;=\;\sqrt{\frac{\left(n_{1}\;-\;1\right)\;SD_{1}^{2}\;+\;\left(n_{2}\;-\;1\right)\;SD_{2}^{2}}{n_{1}\;+\;n_{2}\;-\;2}} \end{array}$$

where *n*_Non−Deprived_ and *n*_Deprived_ are the sample sizes for the non-deprived and deprived groups, respectively, and *SD*_Non−Deprived_ and *SD*_Deprived_ are the standard deviations for the non-deprived and deprived groups, respectively. For within-subject designs, the pooled *SD* was calculated using Equation (3),


(3) $$\begin{array}{l} {Pooled\;SD}_{Within}\;=\;\sqrt{\frac{SD_{1}^{2}\;+\;SD_{2}^{2}}{2}} \end{array}$$

where *SD*_Non−Deprived_ and *SD*_Deprived_ are the standard deviations from the non-deprived and deprived states, respectively. A small subset of studies reported standard errors only; therefore, we calculated standard deviations for these studies by multiplying the standard error by √*n*. For studies that did not report descriptive statistics, we calculated Cohen's *d* from paired-samples *t*-statistics using Equation (4),


(4) $$\begin{array}{l} \text{Cohen’s d}=\frac{t}{\sqrt{n}} \end{array}$$

After obtaining Cohen's *d* for each study, we calculated Hedge's *g* with Equation (5),


(5) $$\begin{array}{l} \text{Hedge’s g}=\text{Cohen’s d}\times \;J \end{array}$$

where *J* is a correction applied to Cohen's *d* to correct for an upward bias in *d* (Borenstein et al., 2009). The correction *J* was calculated with Equation (6),


(6) $$\begin{array}{l} J\;=\;\left(1\;-\;\frac{3}{4df\;-\;1}\right) \end{array}$$

where *df* are the degrees of freedom, given by *N* – 2 for between-subject designs and *N*_Pairss_ – 1 for within-subject designs. Finally, we calculated 95% confidence intervals around each Hedge's *g*. To do this, we first calculated the variance of Cohen's *d* for between-subject designs with Equation (7),


(7) $$\begin{array}{l} V_{d}\;=\;\frac{n_{1}\;+\;n_{2}}{n_{1}n_{2}}\;+\;\frac{d^{2}}{2\left(n_{1}\;+\;n_{2}\right)} \end{array}$$

and for within-subject designs with Equation (8),


(8) $$\begin{array}{l} V_{d}\;=\;\left(\frac{1}{n}\;+\;\frac{d^{2}}{2n}\right)2\left(1\;-\;r\right) \end{array}$$

where *r* is correlation between observations in a pair. As in Rung and Madden (2018), we assumed an *r* = 0.5 for all studies. Next, we calculated the variance of Hedge's *g* with Equation (9),


(9) $$\begin{array}{l} V_{g}\;=\;J^{2}\;\times \;V_{d} \end{array}$$

From the variance of Hedge's *g*, we calculated the standard error (*SE*) with Equation (10),


(10) $$\begin{array}{l} SE_{g}\;=\;\sqrt{V_{g}} \end{array}$$

and confidence intervals with Equation (11),


(11) $$\begin{array}{l} 95\%\;C.I.=\text{Hedge’s g}\pm \;\left(1.96\;\times \;SE_{g}\right) \end{array}$$

**Figure 2:**
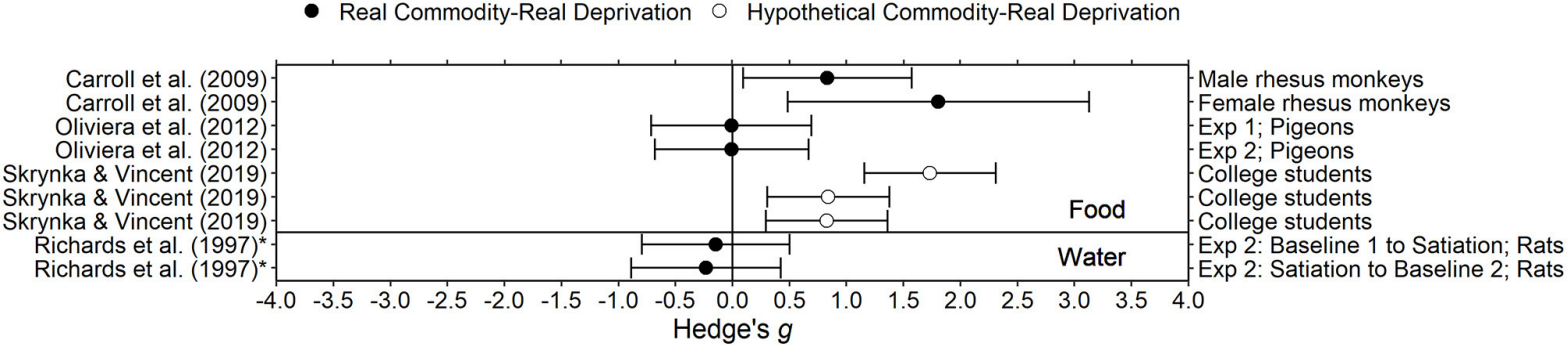
Effect sizes for food and water deprivation experiments. Effect size is Hedge's *g*. Points closer to 0 indicate smaller effect sizes. Points to the right of the line indicate increases in delay discounting due to deprivation, the predicted effect. Solid circles indicate that the delay discounting task used real outcomes and subjects experienced real deprivation. Unfilled circles indicate that the delay discounting task used hypothetical outcomes and subjects experienced real deprivation. *Indicates the effect size was calculated using non-transformed *k*-values and thus may be biased.

**Figure 3:**
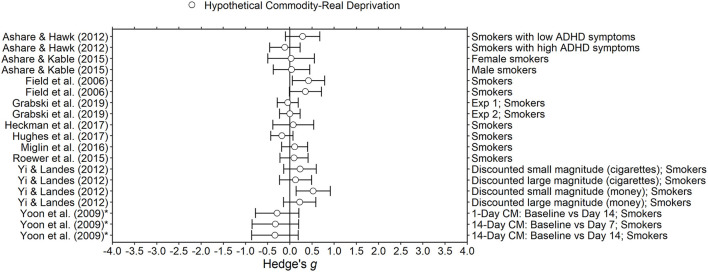
Effect sizes for nicotine deprivation experiments. Effect size is Hedge's *g*. Points closer to 0 indicate smaller effect sizes. Points to the right of the line indicate increases in delay discounting due to deprivation, the predicted effect. Subjects discounted hypothetical outcomes and experienced real deprivation. *Indicates the effect size was calculated using non-transformed k-values and thus may be biased.

**Figure 4:**
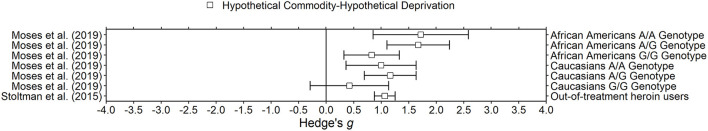
Effect sizes for opioid deprivation experiments. Effect size is Hedge's *g*. Points closer to 0 indicate smaller effect sizes. Points to the right of the line indicate increases in delay discounting due to deprivation, the predicted effect. Subjects discounted hypothetical outcomes and experienced hypothetical deprivation.

**Figure 5:**
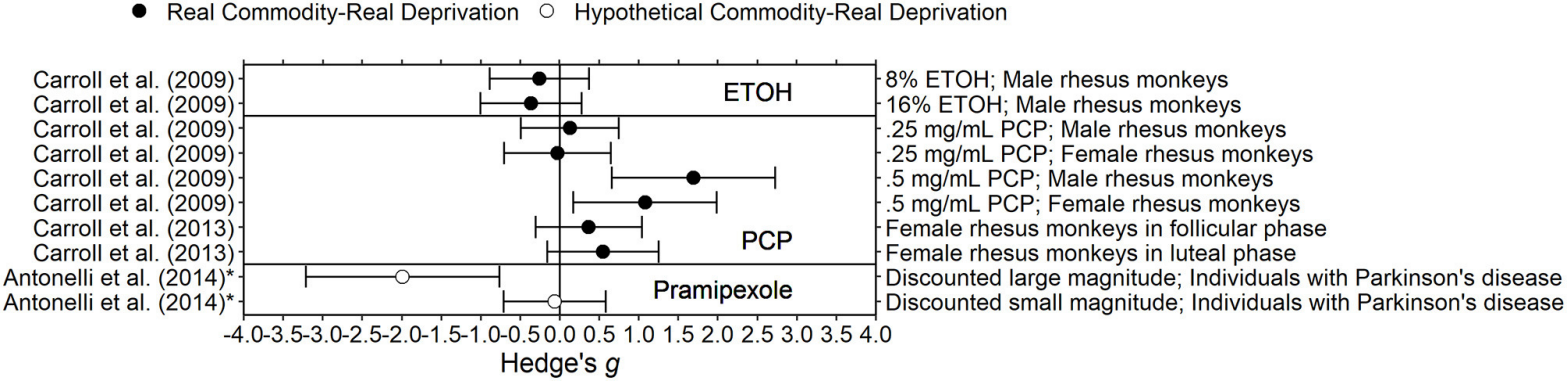
Effect sizes for other drug deprivation experiments. Effect size is Hedge's *g*. Points closer to 0 indicate smaller effect sizes. Points to the right of the line indicate increases in delay discounting due to deprivation, the predicted effect. Solid circles indicate that the delay discounting task used real outcomes and subjects experienced real deprivation. Unfilled circles indicate that the delay discounting task used hypothetical outcomes and subjects experienced real deprivation.

**Figure 6:**
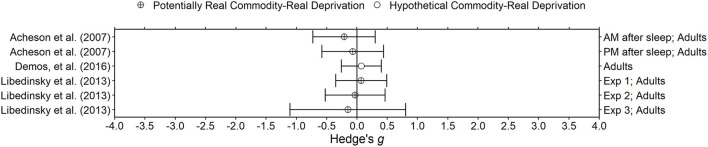
Effect sizes for sleep deprivation experiments. Effect size is Hedge's *g*. Points closer to 0 indicate smaller effect sizes. Points to the right of the line indicate increases in delay discounting due to deprivation, the predicted effect. Circles with a cross indicate that the delay discounting task used potentially real outcomes and subjects experienced real deprivation. Unfilled circles indicate that the delay discounting task used hypothetical outcomes and subjects experienced real deprivation.

**Figure 7:**
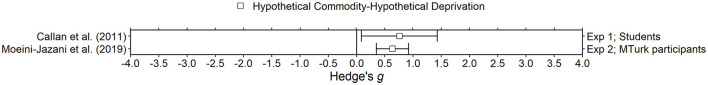
Effect sizes for financial deprivation experiments. Effect size is Hedge's *g*. Points closer to 0 indicate smaller effect sizes. Points to the right of the line indicate increases in delay discounting due to deprivation, the predicted effect. Subjects discounted hypothetical outcomes and experienced hypothetical deprivation.

Figures 2–7 show effect sizes for each study for each outcome type of which subjects were deprived. Both Hedge's *g* and Cohen's *d* are computed using standardized mean differences, and thus interpretation of the two are similar (Ferguson, 2009). The midline represents an effect size of 0, which indicates delay discounting does not differ during deprivation and control conditions. Accordingly, effect sizes farther from the midline are larger. Effect sizes to the left of the midline are negative, and indicate that delay discounting was lower (i.e., less impulsivity) in the deprivation condition than in the control condition (the opposite of the predicted effect). Effect sizes to the right of the midline are positive, and indicate that delay discounting was higher (i.e., more impulsivity) in the deprivation condition than in the control condition (the predicted effect).

## Results and Discussion

Overall, we found inconsistent effects of deprivation on delay discounting. The effect sizes we computed range from Hedge's *g* = −1.98 to 1.81. To try to better understand why the range of effect sizes is so large, we grouped the findings with respect to the outcome of which subjects were deprived. We found that most studies could be classified into the broader deprivation categories of Food and Water Deprivation, Nicotine Deprivation, Opioid Deprivation, Deprivation of Other Drugs, Sleep Deprivation, and Financial Deprivation. For each deprivation type, we also discuss physiological, affective, or cognitive changes that subjects may experience as a result of the deprivation manipulation. We first discuss studies with human and non-human animal subjects, then we discuss studies with only human participants.

### Food and Water Deprivation

Surprisingly, unlike other deprivation manipulations, moderate food and water deprivation have few effects on cognition and behavior. Benau et al. (2014) concluded that short-term fasting in humans has inconsistent or no effects on cognition (e.g., Zajac et al., 2021), but does affect motor performance (reducing reaction times) and increases negative affect. Food restriction in rats may lead to increased operant responding for drugs and to increased levels of corticosterone (i.e., stress; Carroll, 1985; Nowland et al., 2011), but this effect is generally studied as a long-term manipulation rather than a short-term state manipulation. Moderate water deprivation in humans similarly has, in general, no consistent effects on cognition, but does increase negative affect and decrease alertness (e.g., Neave et al., 2001; see Masento et al., 2014, for review). One study with human participants and three studies with non-human animal subjects in the present review examined the effect of deprivation of food or water on delay discounting.

#### Human Participants

Skrynka and Vincent (2019) examined delay discounting of hypothetical money, food, and music in 50 college students. For one session, participants were instructed to eat in the 2 h before coming to the laboratory, and in the other session, participants were instructed to fast for 10 h prior to the session. Manipulation compliance was verified by assessing blood glucose levels and subjective craving in each session. Blood glucose was within normal fasting levels for the majority of participants and subjective craving was significantly higher during the 10 h fast condition compared to the control condition. For the adjusting amount delay discounting task, delays ranged from 1 h to 1 year and the larger later amounts were equivalent to £20. Delay discounting was higher in the deprivation condition compared to the control condition for all commodities. Interestingly, the increase in delay discounting for food (Hedge's *g* = 1.73), an in-domain commodity, was larger than the increase in delay discounting for music downloads and money (Hedge's *g*s = 0.83 and 0.84, respectively), out-domain commodities.

#### Non-human Animal Subjects

In Richards et al. (1997), eight Sprague-Dawley rats discounted 100 μl water while deprived of water and while partially satiated in an ABA design. In the water deprivation condition (A), rats had 20 min of access to water per day, available immediately after a delay discounting session. The satiation condition (B) consisted of an additional 20 min of water available 4 h prior to the delay discounting session. Deprivation resulted in small *decreases* in delay discounting (Hedges *g*s = −0.23, −0.14; the opposite of the predicted direction). Richards et al. (1997) concluded that there is no effect of deprivation of water on delay discounting. Providing support for this conclusion, Richards et al. (1997) suggested that the manipulation was effective in manipulating water deprivation because weight and latency (time to respond in the task) increased with greater access to water.

In Carroll et al. (2009), 8 male and 5 female rhesus monkeys discounted self-administered phencyclidine (PCP) during food restriction and food satiation. Food restriction (i.e., deprivation) was defined as 85% free feeding weight and satiation was defined as being fed double the amount required to maintain 85% of free feeding weight. In the satiation condition, the amount of food was adjusted so that monkeys left at least 100 g of food uneaten. On average, delay discounting was greater during the restriction condition than in the satiation condition for both sexes (Hedge's *g*s = 0.83 for males, 1.81 for females). In a similar experiment (Carroll et al., 2009), male rhesus monkeys (*n* unspecified) also discounted PCP but were deprived of a saccharin solution. In the satiation (non-deprived) condition, saccharin (1,900 mL daily) was available for at least 14 days. In the deprivation condition, water replaced saccharin. Water intake during the saccharin deprivation period was much lower than was saccharin intake during the satiation condition. However, delay discounting was similar during the saccharin deprivation and satiation conditions.

Oliveira et al. (2013) examined the effect of food deprivation on delay discounting in pigeons using two different deprivation procedures. In the first experiment, deprivation was controlled by modulating percentage of free feeding weight: during the deprivation condition 5 female pigeons were maintained at 75–80% free feeding weight, and during the control (i.e., satiation) condition the same pigeons were maintained at 90–95% free feeding weight. In the second experiment, deprivation was controlled by modulating time since the last feeding. In the deprivation condition, six male pigeons were deprived of food for 23 h prior to the delay discounting sessions. In the control condition, the same pigeons were deprived of food for 1 h prior to sessions. In both conditions, pigeons were maintained at 80–85% free feeding weight. For both deprivation procedures, delay discounting during the deprivation condition was not significantly different from delay discounting in the control condition (Hedge's *g*s = −0.01 for Exp. 1, −0.007 for Exp. 2). Only measures for delay discounting were reported; it was not stated whether other behavior changed due to the manipulation.

#### Conclusion

Overall, the effects of deprivation of food and water on delay discounting are inconsistent. One possible limitation in this area is the relatively small sample sizes used; five out of six of the experiments described above used a sample size <15. According to a power analysis, for a two-tailed paired samples *t-*test, a sample size of 90 is required to detect a medium effect size (Cohen's *d* = 0.3) when power is set to 0.8 and the significance level is set to 0.05 (Faul et al., 2007). However, in two studies, the effect size was negative, indicating increased deprivation may have decreased delay discounting, which is in the opposite direction than predicted.

As noted in Skrynka and Vincent (2019), studying deprivation state and measuring delay discounting of different commodities, specifically in- and out-of-domain commodities, may help to demonstrate the extent to which delay discounting is state-like or trait-like. If delay discounting is purely state-like, delay discounting of all outcomes should increase similarly due to a state manipulation, regardless of whether the state manipulation is relevant to the commodity discounted. If delay discounting is somewhere between a state and a trait, then the commodity discounted would play a larger role in determining degree of delay discounting for each commodity (see Figure 1 in Skrynka and Vincent, 2019). This point has implications for manipulations that seek to reduce delay discounting; effective interventions would influence behavior in all domains (i.e., financial, health, social) instead of just one.

### Nicotine Deprivation

Nicotine withdrawal symptoms in humans are somatic, affective, and cognitive. Symptoms include irritability, increased appetite, difficulty paying attention, and impaired working memory (Heishman et al., 2010; McLaughlin et al., 2015). Withdrawal is thought to begin within 3–4 h of abstinence and may last up to 4 weeks (Hughes, 2007; McLaughlin et al., 2015). Impatience and impulsivity have been investigated as symptoms of nicotine withdrawal (Hughes, 2007; Hughes et al., 2014). Although smoking cessation treatments have been developed (Jorenby et al., 2006; Dallery and Raiff, 2011), many smokers trying to quit relapse within about a week (Hughes et al., 2004). Because withdrawal symptoms may play a role in relapse (Robinson et al., 2019), it is important to understand any withdrawal-related changes in cognitive processes, such as delay discounting, that occur during this time (Ashare and McKee, 2012; Ashare et al., 2014). If changes in delay discounting during withdrawal lead to more or less successful quit attempts, modulating delay discounting may help to improve quit outcomes (see Miglin et al., 2017; Rung and Madden, 2018). A total of 13 articles in the present review conducted experiments to determine the effects of nicotine abstinence on delay discounting. Effect sizes ranged from close to 0 (i.e., no change in delay discounting; Hedge's *g* = −0.04) to large and positive (Hedge's *g* = 0.64; see Figure 4). Several factors may explain differences between results including the samples, the deprivation length, and delay discounting tasks. Of the 13 nicotine deprivation articles, 11 used human participants.

#### Human Participants

In all 11 human experiments, deprivation from nicotine was verified biochemically and with subjective assessments. Biochemical abstinence was verified in all studies by analyzing expired carbon monoxide (CO) breath content. The maximum ppm allowed for abstinence varied from 4 to 11 ppm. Although analyzing CO breath content does provide indication of acute abstinence, it may not be able to verify complete abstinence over the entire 24 h deprivation periods that many studies used (Jatlow et al., 2008). Three studies analyzed cotinine content from urinalyses, which allows for detection of all nicotine consumption, rather than just inhaled, over a longer period (Haufroid and Lison, 1998; Jatlow et al., 2008). The two studies with the longest deprivation periods used urine cotinine analysis, providing confidence that participants did indeed maintain abstinence for weeks. Both studies (Yoon et al., 2009; Hughes et al., 2017) found no change in delay discounting during nicotine abstinence. The deprivation manipulations were also verified with cravings and withdrawal symptom assessments, the most common ones being the Questionnaire on Smoking Urges (QSU; Tiffany and Drobes, 1991) and the Minnesota Nicotine Withdrawal Scale (MNWS; Hughes and Hatsukami, 1986). All 11 studies included some form of either the QSU or MNWS. Expired CO was lower, and cravings and withdrawal symptoms were higher, in nicotine deprivation sessions compared to satiated sessions for all studies that made this comparison. Finally, many studies included a battery of tasks in addition to delay discounting tasks. Changes in other tasks (e.g., cross-commodity discounting, time reproduction task, response time) were observed during deprivation for all studies that found no change in delay discounting during deprivation. For example, Ashare and Kable (2015) found no effect of nicotine deprivation on delay discounting but did find that accuracy in a time discrimination task was lower during deprived sessions compared to satiated sessions. This combined evidence suggests that overall, deprivation manipulations were effective and produced changes in deprivation state, providing increased confidence in the results.

Overall, experimental design was relatively similar across studies. All but one study (Heckman et al., 2017) made within-subject comparisons. Participants completed delay discounting tasks about 1 week apart except in one study, Roewer et al. (2015), in which the time between sessions was 24 h. Three studies were contingency management studies; participants were paid for biochemically verified abstinence (Yoon et al., 2009; Hughes et al., 2017; Miglin et al., 2017). In Yoon et al. (2009) and Hughes et al. (2017), participants completed delay discounting tasks more than two times and remained in the study for at least 2 weeks. In both studies, delay discounting remained relatively stable over time. Recall that withdrawal symptoms may last up to 4 weeks (Hughes, 2007; McLaughlin et al., 2015). Both studies were measuring delay discounting during times nicotine withdrawal symptoms have been observed previously. Although both of the longer contingency management studies (Yoon et al., 2009; Hughes et al., 2017) showed no increase in delay discounting over time, there was no consistent pattern for shorter deprivation lengths. Field et al. (2006) and Heckman et al. (2017) used similar lengths of at least 12 and 13 h, respectively, and reported increased delay discounting, whereas Ashare and McKee (2012) and Grabski et al. (2020) also used shorter deprivation lengths (<24 h) but found no effect of deprivation. The most common deprivation length was 24 h (*n* = 5). All but one study, Yi and Landes (2012), found no effect of 24 h of deprivation on delay discounting. Future research could examine whether delay discounting fluctuates systematically during the first few days of abstinence.

Studies on the effect of nicotine deprivation on delay discounting used markedly different samples (see Table 1). Mean age varied from 20 to 45 years across studies; some samples were college students, and some were community members. Because mean age differed by more than 20 years across studies, maximum length of nicotine dependence necessarily differed. Mean score on the Fagerstrom Test for Nicotine Dependence (FTND; Heatherton et al., 1991) ranged from 3.57 to 7.24 out of a maximum of 10, indicating large differences between level of nicotine dependence. Study requirements for number of cigarettes smoked per day also varied from 5 (Grabski et al., 2020) to 25 (Roewer et al., 2015). Mean number of cigarettes smoked ranged from 11 to more than 25 per day. Sample size ranged from 11 to 67 for within-subject comparisons, indicating that power to detect differences between conditions also varied greatly. Two studies required participants to be trying to quit and seven studies specifically excluded smokers trying to quit. We found no clear relationship between studies with larger effect sizes and participant age, dependence, or daily cigarettes smoked.

**Table 1:** Comparison of participant characteristics and delay discounting tasks in nicotine deprivation experiments.

				**Participant characteristics**					**Delay discounting task**		
**References**	**Deprivation length**	**Effect size**	**Author conclusion**	***n***	**Age**	**FTND**	**Cig. per day**	**Quit status**	**Longest delay**	**Outcome**	**Magnitude**
Mitchell (2004)	24 h	—	No effect	11	20.2	5^a^	18.9	—	365 days	Potentially real money	LL $10
Field et al. (2006)	≥13 h	0.42	Increase	30	23.3	3.6	15	Not trying to quit	25 years	Hypothetical money	LL 500 £
”	”	0.35	”	”	”	”	”	”	”	Hypothetical cigarettes	LL 500 £ worth of cigarettes
Ashare and Hawk (2012)	Overnight	0.29	Increase (Low ADHD group)	25	44	5.2	20	Not trying to quit	180 days	Hypothetical money	LL $100
”	”	−0.11	No effect (High ADHD group)	31	37	5.3	17	Not trying to quit	”	”	”
Ashare and McKee (2012)	≥18 h	—	No effect	58	35.9	5.6	18.7	Not trying to quit	179 days	Hypothetical money	$25–$85
Yi and Landes (2012)	24 h	0.64	Increase	28	40	6.4^a^	21	Not trying to quit	10 years	Hypothetical money	LL $50 and $1,000
”	”	0.22	No effect	”	”	”	”	”	10 years	Hypothetical cigarettes	LL $50 and $1,000 worth of cigarettes
”	”	—	No effect	”	”	”	”	”	6 months	Potentially real money	LL $50
Roewer et al. (2015)	24 h	0.09	No effect	37	33	7.2	≥ 25	—	190 days	Hypothetical money	SS $10
Ashare and Kable (2015)	24 h	0.07	No effect (Male)	21	37.1	4.6	18.6	Not trying to quit	months	Hypothetical money	—
“	”	0	No effect (Female)	12	40.2	4.8	14.3	”	”	”	”
Heckman et al. (2017)	12 h	0.09	Increase	128	37	6	20	Not trying to quit	179 days	Hypothetical money	$25–$85
Miglin et al. (2017)	24 h	0.11	No effect	43	45	4.9	13.7	Trying to quit	174 days	Hypothetical money	$15–$85
Grabski et al. (2020)	≥8 h	−0.05	No effect	67	21.8	4.4	11	Not trying to quit	365 days	Hypothetical money	LL 100 £
Hughes et al. (2017)	4 weeks^b^	−0.18	Decrease	61	40	5	19	Trying to quit	5 years	Hypothetical money	LL $1,000
Yoon et al. (2009)	<24 h	−0.29	No effect	15	28.1	5.3	18.2	Not trying to quit	25 years	Hypothetical money	LL $1,000
”	7 days	−0.33	”	13	29.1	6.2	21.7	”	”	”	”
”	14 days	−0.34	”	”	”	”	”	”	”	”	”

*Effect size is Hedge's g. FTND, Fagerstrom Test for Nicotine Dependence. Means are listed for age, FTND score and cigarettes per day. —Indicates information was not specified. “Indicates the cell contains the same information as the cell above. Author conclusion refers to conclusion made about the effect of nicotine deprivation on delay discounting by authors of the original study, not our conclusion. LL refers to the larger later amount used in the delay discounting task*.

a *Fagerstrom Tolerance Questionnaire*.

b *Delay discounting was measured 8 times over 4 weeks. This effect size compares average delay discounting at baseline to average delay discounting over 4 weeks of abstinence*.

Older and younger smokers, more and less dependent smokers, and smokers trying or not trying to quit may differ in important ways that make comparisons between studies difficult or even inappropriate. Some studies were also published over a decade apart; a sample of smokers in 2004 may be different in important ways from a sample of smokers in 2020 (Hughes, 2011; Drope et al., 2018; Grant et al., 2020). However, because studies examined many types of smokers and made similar conclusions for different types of smokers, the findings are more general.

Variations in delay discounting tasks may also have contributed to the discrepancy between results (see Table 1). Delay discounting has been shown to be generally similar regardless of real or hypothetical outcomes (Johnson and Bickel, 2002; Madden et al., 2003). Interestingly, all studies that included a potentially real outcome task found no change in delay discounting of potentially real money due to deprivation (Mitchell, 2004; Yi and Landes, 2012; Roewer et al., 2015). Some authors have suggested that tasks with experienced delays and outcomes may be required to see the effect of state manipulations (e.g., Reynolds and Schiffbauer, 2004; Dallery and Raiff, 2007), but the effect sizes computed in the current review for other deprivation types may indicate otherwise. However, all potentially real outcomes in these studies were necessarily small amounts of money, which means that comparisons between results for real and hypothetical outcomes may be confounded by the amount of the outcome.

Although we did not compute effect sizes for cross commodity tasks, during deprived states, Mitchell (2004) found *increased* preference for immediate cigarettes over delayed money whereas Yoon et al. (2009) found *decreased* preference for immediate cigarettes. Both Mitchell (2004) and Yoon et al. (2009) suggest that the change in the reinforcing value of the outcome, rather than changes in sensitivity to delay, may play a role in the changed cross-commodity discounting. In Mitchell (2004), participants were required to stay in the laboratory for several hours after their 24 h deprivation period and could smoke only cigarettes earned in the potentially real commodity discounting task, which may have increased immediate desire for cigarettes. The possibility of immediate relief from withdrawal in Mitchell (2004) may have increased the value of cigarettes. In contrast, smokers in Yoon et al. (2009) were paid for several days of abstinence and the value of immediate cigarettes may have decreased due to an increased motivation to quit smoking.

Out of these 11 human-subject studies, only four concluded that nicotine deprivation increased delay discounting (Ashare and Hawk, 2012, in one group only; Field et al., 2006; Heckman et al., 2017; Yi and Landes, 2012, in monetary task only). Field et al. (2006) and Yi and Landes (2012) both found medium to large increases in hypothetical monetary delay discounting (Hedge's *g*s = 0.64 and 0.42, respectively). Ashare and Hawk (2012) found increases in delay discounting in participants with fewer ADHD symptoms, but not in participants with more ADHD symptoms (Hedge's *g* = 0.29). Mean participant age and nicotine dependence, and deprivation duration varied across the studies.). The six studies that found small or no effect of deprivation on delay discounting all had participant *N*'s as large or larger than those with large effect sizes (i.e., Field et al., 2006; Ashare and Hawk, 2012; Yi and Landes, 2012). Additionally, out of the 17 effect sizes we computed for experiments that examined nicotine deprivation, all but two have confidence intervals that overlap with 0 (see Figure 4). For these reasons, we conclude that the effect of acute nicotine deprivation on delay discounting in humans is probably small at most. This conclusion is valid only supposing that delay discounting tasks are indeed sensitive enough to detect pharmacological state changes (Odum and Baumann, 2010; Odum et al., 2020; see, however, De Wit and Mitchell, 2010) and accepting the previously discussed evidence that deprivation actually induced withdrawal in participants.

#### Non-human Animal Subjects

Two articles in the present review examined the effect of nicotine deprivation on impulsive choice in rats. Nicotine withdrawal in rats includes somatic signs such as head and body shakes, teeth chattering, ptosis, and yawns; and may include cognitive changes such as deficits in attention and working memory (Malin et al., 1992; Shoaib and Bizarro, 2005; Ashare et al., 2014). In Kayir et al. (2014), 22 male Wistar rats received 6.32 mg/kg/day of nicotine via an osmotic mini-pump for 13 days. In Kolokotroni et al. (2014), 29 male Lister hooded rats received 3.16 mg/kg/day of nicotine via a mini-pump for 7 days. Rats were food deprived during the duration of both experiments. Impulsive choice tasks were based on Evenden and Ryan (1996); rats were offered 1 pellet immediately and 4 or 5 pellets after delays ranging from 0 to 60 s. Rats were placed into high and low impulsive groups determined by baseline level of impulsivity. Both studies concluded that in low impulsive rats, nicotine deprivation leads to increased choice for smaller sooner food. In high impulsive rats, Kolokotroni et al. (2014) found decreases in choice for smaller sooner food and Kayir et al. (2014) found no change in choice for smaller sooner food. When considering all rats in the study, Kayir et al. (2014) found no change in choice for smaller sooner food during nicotine withdrawal. Important to note, Kayir et al. considered measurements of choice during the first 48 h after pump removal and Kolokotroni et al. (2014) measured choice for weeks after pump removal and found effects of withdrawal only during the first week. The results for the high impulsive rats could be explained by a ceiling effect; number of choices for smaller sooner food could have been so high at baseline that there would be less room to increase during deprivation. Or, perhaps, rate dependency may help to explain the difference between high and low impulsive groups (Quisenberry et al., 2016). It could also be that there is in fact a difference in the effects of withdrawal between low and high impulsive rats. In humans, Ashare and Hawk (2012) found a similar effect; those with low ADHD symptoms had greater increases in delay discounting after nicotine abstinence compared to those with high ADHD symptoms. Individual differences in response to nicotine deprivation conditions may help to explain why many other human experiments report no effect of deprivation on delay discounting. It could be that only certain individuals discount differently due to nicotine deprivation; by aggregating data, the effect of deprivation could be averaged away, resulting in apparently no change in delay discounting. Nonetheless, it may be valuable to analyze individual responses to deprivation and other state manipulations, rather than the differences in means across conditions.

### Opioid Deprivation

In people who have opioid dependency, opioid deprivation can lead to pronounced opioid withdrawal symptoms. The severity and onset of opioid withdrawal symptoms depends on the severity of opioid dependence as well as if the opioids last used were short or long-acting (Wesson and Ling, 2003; Kosten and Baxter, 2019). Deprivation of short-acting opioids, including heroin and oxycodone, results in opioid withdrawal symptoms after ~12 h. Symptoms may peak in severity around 36–72 h and then tend to end after 4–7 days (Kosten and Baxter, 2019). In contrast, opioid withdrawal symptoms for long-acting opioids, including methadone and buprenorphine, may last for 2 weeks (Kosten and Baxter, 2019). Symptom severity is greater for those that are more dependent (Wesson and Ling, 2003), but the same symptoms are seen in users of long- and short- acting opioids (Kosten and Baxter, 2019). Opioid withdrawal symptoms may include anxiety, insomnia, irritability, and cold and flu-like symptoms (i.e., hot and cold flashes, aches, nausea, vomiting, runny nose; Wesson and Ling, 2003; Kosten and Baxter, 2019). Both chronic and acute opioid use are known to produce a range of cognitive impairments (Ersek et al., 2004; Baldacchino et al., 2012). For instance, there is evidence that opioid users tend to discount delayed rewards more steeply than controls (MacKillop et al., 2011). Less is known, however, about specific cognitive changes during acute opioid withdrawal in humans. In the present review, three studies with human participants and one with non-human animal subjects examined the effect of opioid deprivation on delay discounting.

#### Human Participants

In Giordano et al. (2002), 13 participants in outpatient treatment for opioid dependence completed delay discounting tasks 2 h after buprenorphine administration (satiation) and 5 days after buprenorphine administration, when the maintenance dose had worn off (withdrawal). Each condition was repeated 4 times over a period of 8 weeks. Participants were on average 37.5 years old, used 5 bags of heroin daily, and were dependent for 11.9 years. Abstinence from all opioids was verified with urinalysis two to three times per week. Two positive tests for opioids over the course of the 8 week study resulted in discontinuation. An additional 13 participants started the study but did not continue due to failure to provide negative urine samples or failure to return after intake. Withdrawal was assessed with pupil radius measures and with subjective assessments. Subjective assessments of withdrawal and pupil radiuses were significantly higher during withdrawal compared to satiated conditions. Adjusting amount delay discounting tasks used outcomes of money and number of bags of heroin at magnitudes of $100, 3,000, and 10,000 (equivalent worth for bags of heroin). For the 13 participants that completed the study, *k*-values were significantly higher for deprived conditions compared to sated conditions for all commodity and magnitude combinations. Giordano et al. (2002) results demonstrated that among opiate-dependent individuals, opioid deprivation may substantially increase delay discounting. It should be noted, however, that the sample size was small, and the experiment had a high attrition rate. Thus, the results should be considered with caution. Two experiments that employed hypothetical opioid deprivation may help to provide additional evidence of an increase in delay discounting during opioid deprivation.

Stoltman et al. (2015) and Moses et al. (2019) developed a hypothetical opioid deprivation model and found that delay discounting was steeper during deprived states than during satiated states. In the hypothetical withdrawal condition, participants were instructed to answer as if they were going through opioid withdrawal. In the satiation condition, participants were instructed to answer as if they had just taken heroin. For both conditions, a few symptoms or feelings associated with the state were given in the oral instructions. The satiated and withdrawal conditions were completed back-to-back and were counterbalanced across participants. The delay discounting task was developed to be more ecologically relevant to decisions heroin users might regularly face; the delays ranged from 3 to 96 h and the larger later amount was 30 bags of $10 worth of heroin. Both studies used relatively large samples (>100) of out of treatment heroin users and required a positive urinalysis to participate. Although both Stoltman et al. (2015) and Moses et al. (2019) found increases in delay discounting, imagined withdrawal is arguably different from experienced withdrawal. It is also unclear if the increase in discounting found in Stoltman et al. (2015) and Moses et al. (2019) would generalize to more traditional delay discounting tasks with larger amounts of money and longer delays. That is, the large effect found due to hypothetical opioid withdrawal may only be large because the task involved heroin. One would predict that delay discounting for opioids and money would be related (Odum et al., 2020) but it is possible that there is an interaction between the deprivation state and the commodity discounted that does not follow the trait-like pattern (i.e., opioid deprivation may produce larger changes in delay discounting of opioids than in delay discounting of money). Nevertheless, all three human studies report increases in delay discounting due to opioid deprivation.

#### Non-human Animal Subjects

In Eppolito et al. (2013), six unsexed pigeons completed impulsive choice tasks with food during daily morphine administration and after morphine discontinuation. For four pigeons, trial omissions increased sharply after discontinuation, limiting the authors' ability to construct delay discounting curves. Interestingly, the number of choices for the larger later amount increased after discontinuation compared to during daily morphine treatment. Despite 8 weeks of, at its highest, 2 daily 100 mg/kg doses of morphine, not all pigeons showed withdrawal signs during saline probes.

In Harvey-Lewis et al. (2015), male Long-Evans rats maintained on subcutaneously injected 30 mg/kg daily morphine doses completed impulsive choice tasks with sucrose during a baseline, satiated condition and 1 h after naloxone-precipitated withdrawal. Naloxone is an opioid antagonist that has been shown to induce withdrawal in rats. Preference for smaller sooner sucrose increased after naloxone administration only for short delays (i.e., 5 and 9 s). The change in number of choices for smaller sooner sucrose depended on the naloxone dose administered, with the larger dose producing greater increases in number of choices for smaller sooner sucrose compared to the smaller dose. Although different doses of naloxone produced significantly different number of choices for smaller sooner sucrose at some delays, the authors did not report a statistical result for the comparison between baseline impulsive choice and impulsive choice after naloxone administration. The authors did, however, conclude that for short delays, naloxone-precipitated withdrawal increased the number of smaller sooner choices.

#### Conclusion

Although four of the five studies reported increases in delay discounting due to opioid deprivation, each had some limitations. Future studies could attempt to further validate and generalize the hypothetical deprivation condition described in Stoltman et al. (2015) and Moses et al. (2019). Once validated, the hypothetical deprivation model could be a preferable alternative to asking participants to voluntarily go through actual opioid withdrawal.

### Deprivation of Other Drugs

Researchers have also examined the effects of deprivation of amphetamine, caffeine, ethanol, PCP, and pramipexole on delay discounting. Due to the limited number of studies in each drug category, we do not draw any general conclusions.

#### Pramipexole

In Antonelli et al. (2014), 7 Parkinson's disease patients completed a delay discounting task (Kirby et al., 1999) after 12–18 h of being deprived of their usual antiparkinsonian medication and then after 1 mg of pramipexole was administered (i.e., satiation). These sessions occurred during the same day and in the same order for each participant. It was not clear if withdrawal signs and symptoms were measured. Delay discounting was significantly higher after pramipexole administration than after deprivation only for the large magnitude task (600–1,000 CAD; Hedge's *g*s = −1.99 for large magnitude, −0.07 for small magnitude).

#### Stimulants

In Gipson and Bardo (2009), 24 male Sprague-Dawley rats self-administered amphetamine (0.03 or 0.1 mg/kg/infusion) for 1 h or 6 h for 36 days. Sucrose delay discounting tasks occurred during a baseline condition, during self-administration, and during 7 days after discontinuation. Compared to baseline, delay discounting increased (i.e., got steeper) during self-administration for rats in the 6 h access group and decreased for rats in the 1 h access group. Over the 7 days after amphetamine discontinuation, delay discounting decreased for rats in the 6 h access group and increased for rats in the 1 h access group, thus resulting in both groups returning to baseline levels of delay discounting. It is not clear if the difference in delay discounting between the first few days of withdrawal were significantly different from the 3 days of the baseline condition.

In Diller et al. (2008), seven male Sprague-Dawley rats received 30 mg/kg per day of caffeine via intraperitoneal injection for at least 15 days. Delay discounting sessions occurred during a control condition, during chronic caffeine administration (i.e., satiation), and during chronic saline administration (i.e., deprivation). AUC was significantly higher (i.e., discounting was less steep) during chronic caffeine administration compared to chronic saline administration. The length of the chronic saline condition was not the same for all rats (mean = 16.2 days). It is not clear if all sessions or a particular subset of sessions was used to determine average delay discounting during saline administration for each rat. There may be an underestimation of the effect if all sessions were used; the effect of deprivation may be different on the first day after withdrawal than the effects of deprivation on day 15 after withdrawal.

#### PCP

In Carroll et al. (2009, 2013) rhesus monkeys self-administered 0.25 or 0.5 mg/mL of PCP for 2 h per day for at least 10 days and discounted saccharin during a baseline condition, self-administration of PCP, and for 6 days after withdrawal of PCP. Carroll et al. (2009) found that for eight males and six females, for both doses, delay discounting of saccharin was steeper during PCP withdrawal compared to baseline (before PCP administration). Only the comparison for males at the 0.5 mg/mL dose was significantly different from baseline, although all dose and gender combinations were in the same direction. Carroll et al. (2013) found that for seven females, for both doses of PCP, delay discounting was steeper during PCP withdrawal compared to baseline, although the magnitude of the effect may have depended on phase of the menstrual cycle.

#### Ethanol

In Carroll et al. (2009), eight male rhesus monkeys self-administered ethanol (8 or 16% wt/vol) for 10 days and discounted saccharin during a baseline condition, self-administration of ethanol, and for 6 days after withdrawal of ethanol. For both doses, delay discounting was not significantly different during ethanol withdrawal compared to baseline.

### Sleep Deprivation

Acute sleep loss has been associated with a variety of physiological and affective changes including decreased positive mood states, increased food intake, and increased blood glucose levels (Landolt et al., 2014). Sleep deprivation may also impair cognitive function, including working memory, attention, and psychomotor tasks (Killgore, 2010; Landolt et al., 2014).

Three studies in the present review examine the effects of sleep deprivation on delay discounting in human participants. Acheson et al. (2007) and Libedinsky et al. (2013) both examined the effect of 24 h of sleep deprivation on delay discounting of potentially real money. In both studies, the 30 or less participants were on average in their early 20 s. Demos et al. (2016) examined the effect of partial sleep deprivation, defined as four nights of 6 h of sleep, using a hypothetical monetary delay discounting task (i.e., Kirby et al., 1999). Demos et al. (2016) used a slightly larger (*n* = 34) and older sample (mean age = 37 years). In all three studies, participants were only included if they had good sleeping habits. All studies used a within-subjects design with 1 week in between sessions, except for Experiment 3 in Libedinsky et al. (2013), which used a between-subjects design. Libedinsky et al. (2013) and Demos et al. (2016) used activity monitors to verify compliance with the sleep manipulations, whereas the sleep deprivation occurred entirely in the laboratory in Acheson et al. (2007). All studies used other measures besides delay discounting and found some differences due to sleep deprivation (e.g., decreases in positive mood, more errors in the Go/No-Go task, increased effort discounting), providing evidence of the effectiveness of the deprivation manipulation. All three studies (six deprived/non-deprived comparisons total) found no effect of sleep deprivation on delay discounting (Hedge's *g*s between −0.21 and 0.07). Although the results of the included studies are consistent, it is possible that a longer sleep deprivation period may induce changes in delay discounting (Libedinsky et al., 2013). Interestingly, the results of the studies in the present review are not consistent with Reynolds and Schiffbauer (2004). They developed an experiential discounting task (EDT), which includes choices involving both delay and probability, and found increases in impulsive choice after participants experienced 21 h without sleep. It may be that the probabilistic aspect of outcomes in the EDT contributed to the increase in impulsive choice; other research has demonstrated increases in risky choices due to sleep deprivation (Killgore, 2010).

### Financial Deprivation

Personal relative deprivation, or more broadly, financial deprivation, can be described as feelings of having fewer monetary resources, especially when compared to others (Moeini-Jazani et al., 2019). People who have been made to feel as if they have fewer financial resources have been shown to consume more calorie-dense food (Briers and Laporte, 2013), purchase more lottery tickets (Haisley et al., 2008), and save less, all arguably present-oriented behaviors (Shah et al., 2012). Lower income is associated with greater risk aversion and elevated delay discounting (Green et al., 1996; Haushofer and Fehr, 2014). It may be that people in financial deprivation states, either actual or experimentally induced, shift their attention to the present, thereby increasing delay discounting (Shah et al., 2012; Moeini-Jazani et al., 2019). An alternative view is that individuals with less money should value monetary outcomes more so than wealthy individuals, thereby leading to a magnitude effect in which wealthy individuals discount more steeply than lower-income individuals because money is less valuable to those with high income (Oliveira et al., 2013).

Three studies in the present review manipulated feelings of financial status in between-subjects designs. Though the three studies use different terminology, they all arguably manipulate the same thing. In Callan et al. (2011; Study 1) participants were told that their discretionary income was about the same or much lower than others (i.e., false feedback) to invoke feelings of relative deprivation. Van den Bergh et al. (2008; Study 3, control group only^1^) manipulated the scale in which participants reported their income and Moeini-Jazani et al. (2019; Study 2, control group only^2^) used versions of both methods with each participant. The scale manipulation method has been established as an effective way to induce feelings of financial deprivation and has been shown to affect performance in other tasks (e.g., Haisley et al., 2008; Briers and Laporte, 2013). Moeini-Jazani et al. (2019) also conducted a pretest to validate their manipulation. Participants in Van den Bergh et al. (2008) and Callan et al. (2011) were students, with a mean age of around 19 years, while participants in Moeini-Jazani et al. (2019) were older (mean age = 36 years) and recruited online via MTurk. Also important to note, the sample size in Moeini-Jazani et al. (2019) was much larger (*n* > 100 for each group) than in Van den Bergh et al. (2008) and Callan et al. (2011; *N*'s ~30–35 for each group). Van den Bergh et al. (2008) and Moeini-Jazani et al. (2019) used fill in the blank delay discounting tasks (e.g., $65 now is worth ____ in *x* months) with a relatively short set of delays (maximum delays were 18 months and 1 month, respectively) and relatively small magnitudes of larger later amounts ($65 and €15, respectively). Callan et al. (2011) used an adjusting amount task with a fixed larger later outcome of $1,000 and a slightly longer maximum delay of 2 years.

For delay discounting of money, all studies found higher levels of delay discounting for those in the deprivation group compared to the non-deprivation group. The effect was large and statistically significant in Callan et al. (2011; Hedge's *g* = 0.76) and Moeini-Jazani et al. (2019; Hedge's *g* = 0.64), but Van den Bergh et al. (2008) did not report any statistical test results for this comparison. Van den Bergh et al. (2008) also examined delay discounting of bars of candy and cans of soda. Mean AUC for the deprived group was similar to the non-deprived group for delay discounting of both candy and soda. Because of the consistency in the data overall, we conclude that delay discounting of money tends to increase after monetary deprivation manipulations. It is unclear how long the effect of the manipulation lasts; the delay discounting task occurred soon after the manipulation in all studies. It is also unclear if the effect would generalize to delay discounting of other commodities, but it is potentially important that participants were discounting a commodity that was in-domain relative to the manipulation (i.e., deprived of money and discounted money).

## Conclusion

We were not able to make conclusions for each deprivation category, but it does appear that the effect of deprivation on delay discounting may depend on the type of deprivation subjects faced. In humans, nicotine and sleep deprivation tend to have little to no effect on delay discounting, whereas opioid deprivation and feelings of financial deprivation tend to increase delay discounting. The effect of deprivation of food and water on delay discounting is less clear. It is interesting that even though theoretical frameworks (e.g., CNDS model) predict increases in delay discounting, we do not see consistent effects for all types of deprivation.

Previous research indicates that delay discounting is both state-like and trait-like (Odum et al., 2020). The inconsistent effects of deprivation on delay discounting may provide additional evidence that delay discounting is not entirely a trait. If delay discounting was purely trait-like, delay discounting would not change due to any deprivation manipulation (see Skrynka and Vincent, 2019). Yet, in the present review, deprivation resulted in increased, decreased, and no change in delay discounting. Although these findings do provide additional evidence that modulation of delay discounting due to state is possible, it is puzzling that not all types of deprivation manipulations resulted in changes in delay discounting.

One interesting pattern we found was that manipulations that were imagined (i.e., imagined opioid withdrawal, financial deprivation) tended to increase delay discounting, whereas manipulations that were more physiological in nature (i.e., sleep and nicotine deprivation) produced little to no change in delay discounting. This result may suggest that the cognitive appraisal of states could propel modulations in delay discounting. That is, intentional acknowledgment of deprivation symptoms may be important in increasing delay discounting. Related, it may also be that the instructions given in imagined state manipulations specifically highlighted a present experience, perhaps thereby shifting attention to the present, similar to how EFT may shift attention to the future (Lin and Epstein, 2014). To test this suggestion, one could examine the effect of imagined sleep deprivation, for example, on delay discounting. All experiments in the present review concluded that actual sleep deprivation had no effect on delay discounting. If imagined sleep deprivation did increase delay discounting, then something about the imagined state, rather than experiencing tiredness, may be causing the change in delay discounting.

Another possible reason we did not see consistent effects of deprivation is that there may be an effect of domain matching. Specifically, manipulations may have a greater impact on delay discounting if the commodity discounted is relevant to the manipulation (see Skrynka and Vincent, 2019). In many of the opioid deprivation and financial deprivation experiments, participants discounted opioids and money, respectively, and we concluded that deprivation tends to increase delay discounting for these deprivation types. We do not consistently see this pattern, however, for other types of deprivation. For instance, of the two studies in which participants discounted cigarettes and were deprived of nicotine, one found increased delay discounting of cigarettes and one found no change in delay discounting of cigarettes (Field et al., 2006; Yi and Landes, 2012). More experiments are needed to examine the potential interaction between domain matching and manipulations; the generalizability of manipulations has implications for behavioral interventions that aim to change delay discounting. It is important to know if changing delay discounting of food will also influence delay discounting of money, for example, and thus if a range of behaviors or just a class of behaviors can be changed by a single intervention.

In dual-systems models, “visceral influences” like hunger and cravings should increase impulsivity (see e.g., Loewenstein, 1996). The types of deprivation we examined all tend to result in some sort of negative cognitive, emotional, or physiologic change, although the severity of deprivation “symptoms” varies. For instance, opioid withdrawal results in flu-like symptoms and nicotine withdrawal may result in irritability and anxiety. Despite all deprivation manipulations resulting in arguably “visceral” states, we do not see consistent effects of deprivation manipulations on delay discounting. It may be that there is a threshold of discomfort or arousal that must be surpassed in order for a visceral influence to result in significant dysregulation of the control and valuation systems, and thus in heightened delay discounting. Nicotine deprivation may certainly be unpleasant, but arguably not as much as opioid deprivation may be.

It may be instead, rather than visceral influences requiring a threshold, that the effect of visceral influences on delay discounting is more nuanced than previously thought. In Richards et al. (1997) and Oliveira et al. (2013), the effects of water and food deprivation were studied in non-human animals; there was little to no effect of deprivation on delay discounting in these studies (although see Carroll et al., 2009). Non-human animals provide arguably more experimental control (e.g., one can be more certain that a non-human animal subject followed the deprivation protocol). If there is little effect of food and water deprivation on delay discounting in non-human animal models, then perhaps our idea of what constitutes a visceral influence should change. However, the non-human animal literature on delay discounting and deprivation must be reconciled with the human literature, as we found some discrepancies in results between species.

The effects of deprivation, and other states, on delay discounting may provide the impetus for the design of behavioral interventions. In the present review, we found that opioid deprivation tends to result in increased delay discounting; it may be that increased delay discounting during abstinence leads to greater relapse vulnerability. By knowing if people tend to discount future outcomes more so while going through withdrawal, contingency management treatments, for instance, could be designed with shorter delays to incentives to leverage preference for sooner outcomes (Miglin et al., 2017). Similarly, EFT has been shown to reduce self-administered cigarette puffs in the laboratory (Stein et al., 2016); perhaps EFT cues could be administered strategically during deprivation states like abstinence to compensate for maladaptive increases in delay discounting.

The present review is the first to examine the effect of experimental manipulations of deprivation on delay discounting. We found more types of deprivation manipulations than we anticipated, and therefore our search terms may have been limited. Despite this limitation, our review provides the advantage of examining the deprivation literature broadly. As more data emerge, it may be fruitful to examine each deprivation type individually with search terms more relevant to specific manipulations (e.g., Hughes et al., 2014).

Deprivation does not always increase delay discounting, contrary to the predictions of theoretical frameworks. Delay discounting may be a trans-disease process and has been used as target for behavioral interventions (Bickel et al., 2019). Thus, a basic understanding of how delay discounting is affected by various states, including deprivation, will be needed for future translational research.

## Data Availability Statement

The datasets presented in this study can be found in online repositories. The names of the repository/repositories and accession number(s) can be found at: https://osf.io/vjb6e/?view_only=96119bfc0dc04bccbd0698808f59621a.

## Author Contributions

All authors were involved in conceptualization and design. HJ, JH, and HD collected the data. JH conducted the data analysis and created figures. HJ designed a figure. HD wrote the manuscript with support from AO and JH. All authors reviewed the results and approved the final version of the manuscript.

## Conflict of Interest

The authors declare that the research was conducted in the absence of any commercial or financial relationships that could be construed as a potential conflict of interest.

## Publisher's Note

All claims expressed in this article are solely those of the authors and do not necessarily represent those of their affiliated organizations, or those of the publisher, the editors and the reviewers. Any product that may be evaluated in this article, or claim that may be made by its manufacturer, is not guaranteed or endorsed by the publisher.

## Footnotes

^1^While Heckman et al. (2017) concluded nicotine deprivation increased delay discounting (Cohen's d = 0.36), we found the effect to be small based on our calculations from the descriptive statistics reported (Cohen's d = 0.06; Hedge's g = 0.08). We believe this finding is because effect sizes computed from F-statistics may be upwardly biased (Hullett and Levine, 2003).

^2^Van den Bergh et al. (2008) and Moeini-Jazani et al. (2019) both had other manipulations hypothesized to interact with monetary deprivation to increase or decrease delay discounting; we look at only the control condition to more clearly understand only the effects of the deprivation manipulation.
